# Supplementary figures and images for: Cloning and high-level expression of monomeric human superoxide dismutase 1 (SOD1) and its interaction with pyrimidine analogs
Source: PLoS One. 2021 Feb 26;16(2):e0247684. doi: 10.1371/journal.pone.0247684 (PMC7909654; doi:10.1371/journal.pone.0247684)

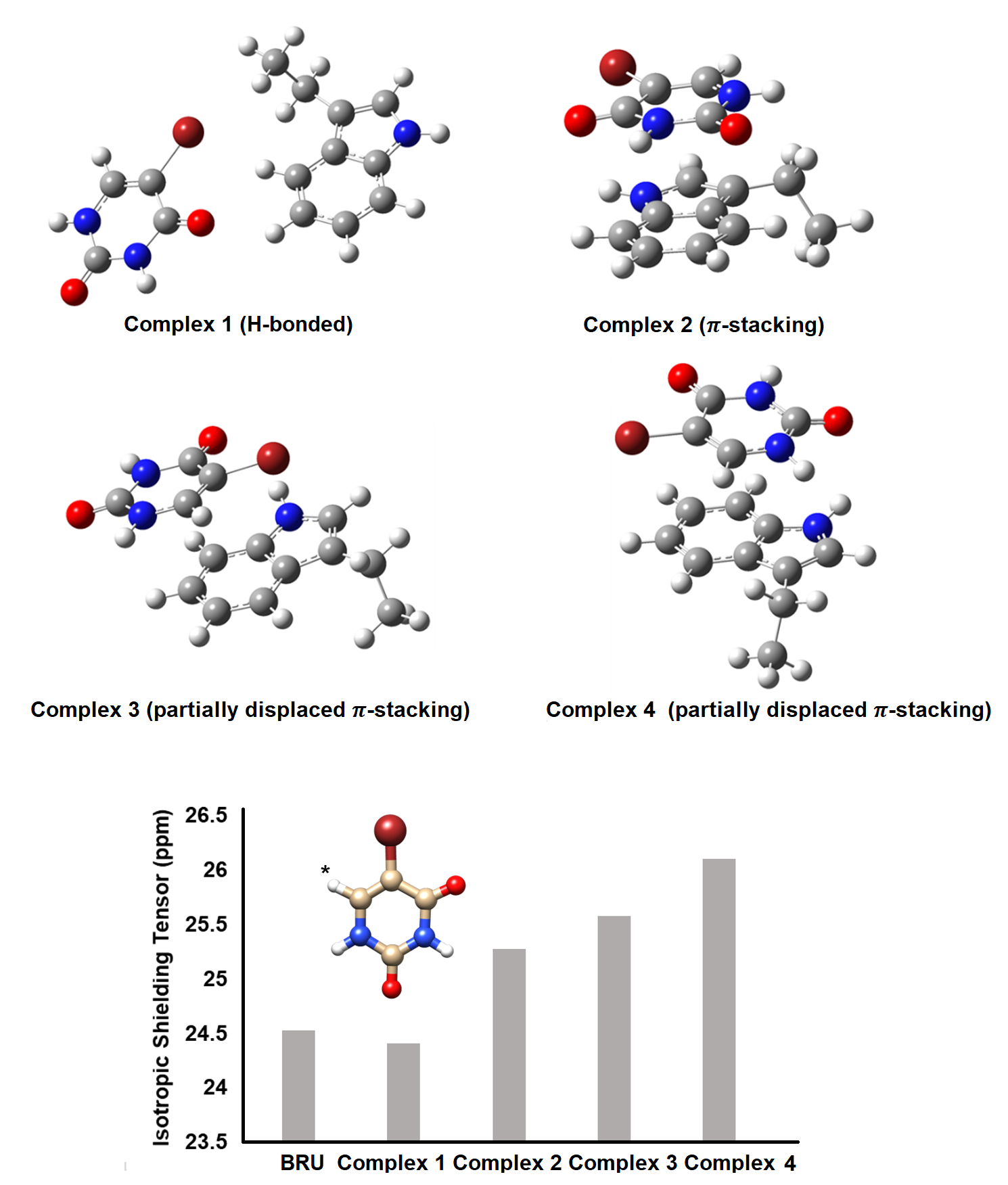

Supplement: S1 Fig — (TIF) [file pone.0247684.s001.tif]
