# Supplementary material for: Cloning and high-level expression of monomeric human superoxide dismutase 1 (SOD1) and its interaction with pyrimidine analogs
Source: PLoS One. 2021 Feb 26;16(2):e0247684. doi: 10.1371/journal.pone.0247684 (PMC7909654; doi:10.1371/journal.pone.0247684)
Supplement: S1 Raw images — (PDF) [file pone.0247684.s002.pdf]

All gels except the bottom two are 12% Tricine SDS-PAGE stained with Coomassie G250. All gels were scanned using a Canon 5600F flatbed scanner using the MP Navigator software and saved as a .jpeg file.

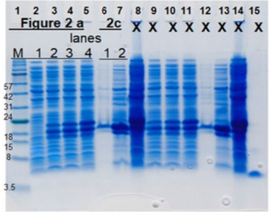

Lanes 2-8 were from cultures grown at RT in LB medium without Cu^{2+} or Zn^{2+}
lanes 9-14 were from cultures grown with Cu^{2+} or Zn^{2+}
1 BLUelf prestained protein ladder
2. Before induction with 1.0 mM IPTG
3. 2hr after induction with 1.0 mM IPTG
4. 4hr after induction with 1.0 mM IPTG
5. after overnight induction with 1.0 mM IPTG
6. sucrose extract after overnight induction with 1.0 mM IPTG
7. periplasmic extract after overnight induction with 1.0 mM IPTG
8. pellet after periplasm was extracted
9. 2hr after induction with 1.0 mM IPTG
10. 4hr after induction with 1.0 mM IPTG
11. after overnight induction with 1.0 mM IPTG
12. sucrose extract after overnight induction with 1.0 mM IPTG
13. periplasmic extract after overnight induction with 1.0 mM IPTG
14. pellet after periplasm was extracted
15. purified Abeta 42 C-terminal 6 lysines

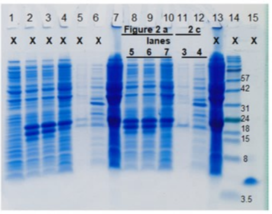

Lanes 2-7 were from cultures grown at 37 °C in LB medium without Cu^{2+} or Zn^{2+}
lanes 8-14 were from cultures grown with Cu^{2+} or Zn^{2+}
1. Before induction with 1.0 mM IPTG
2. 2hr after induction with 1.0 mM IPTG
3. 4hr after induction with 1.0 mM IPTG
4. after overnight induction with 1.0 mM IPTG
5. sucrose extract after overnight induction with 1.0 mM IPTG
6. periplasmic extract after overnight induction with 1.0 mM IPTG
7. pellet after periplasm was extracted
8. 2hr after induction with 1.0 mM IPTG
9. 4hr after induction with 1.0 mM IPTG
10. after overnight induction with 1.0 mM IPTG
11. sucrose extract after overnight induction with 1.0 mM IPTG
12. periplasmic extract after overnight induction with 1.0 mM IPTG
13. pellet after periplasm was extracted
14. BLUelf prestained protein ladder
15. purified Abeta 42 C-terminal 6 lysines

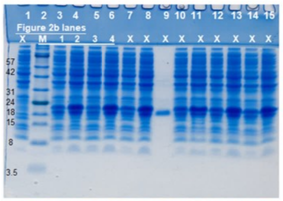

Lanes 1, 3-4, from cultures grown at RT in LB medium without Cu^{2+} or Zn^{2+}
Lanes 5-12 from cultures grown at RT in LB medium with Cu^{2+} or Zn^{2+}
1. Before induction with 1.0 mM IPTG
2. BLUelf prestained protein ladder
3. 2 hr after induction with 1.0 mM IPTG
4. After overnight growth with 1.0 mM IPTG
5. 2 hr after induction with 0.1 mM IPTG
6. After overnight with 0.1 mM IPTG
7. 2 hr after induction with 0.2 mM IPTG
8. After overnight with 0.2 mM IPTG
9. purified His 6X-SOD 1 wild-type
10. 2 hr after induction with 0.4 mM IPTG
11.After overnight with 0.4 mM IPTG
12. 2 hr after induction with 0.8 mM IPTG
13. After overnight with 0.8 mM IPTG
14. 2 hr after induction with 1.0 mM IPTG
15. After overnight with 1.0 mM IPTG

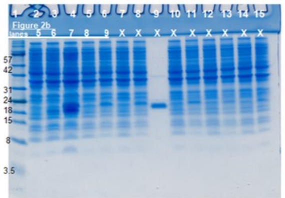

Lanes 2-4, from cultures grown at RT in M9 medium without Cu^{2+} or Zn^{2+}
Lanes 5-15 from cultures grown in M9 medium with Cu^{2+} or Zn^{2+}
1. BLUelf prestained protein ladder
2. Before induction with 1.0 mM IPTG
3. 2 hr after induction with 1.0 mM IPTG
4. After overnight growth with 1.0 mM IPTG
5. 2 hr after induction with 0.1 mM IPTG
6. After overnight growth with 0.1 mM IPTG
7. 2 hr after induction with 0.2 mM IPTG
8. After overnight growth with 0.2 mM IPTG
9. purified His6X-SOD1 wt
10. 2 hr after induction with 0.4 mM IPTG
11. After overnight growth with 0.4 mM IPTG
12. 2 hr after induction with 0.8 mM IPTG
13. After overnight growth with 0.8 mM IPTG
14. 2 hr after induction with 1.0 mM IPTG
15. After overnight growth with 1.0 mM IPTG

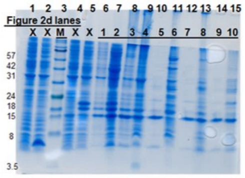

Lanes 1-2 Abeta 42 C6K grown in M9 medium
Lanes 8-15 ammonium sulfate precipitation of SOD1 monomer
1. Abeta $_{42}$ C6K grown in M9 medium at 37 °C before induction with 1.0 mM IPTG
2. Abeta $_{42}$ C6K after overnight growth in M9 medium at 28 °C with 1.0 mM IPTG
3. BLUelf prestained protein ladder
4. SOD1 monomer grown in M9 medium with $^{15}NH_4Cl$ at 37 °C before induction with 0.2 mM IPTG
5. SOD1 monomer after overnight growth in M9 medium with $^{15}NH_4Cl$ at 37 °C with 0.2 mM IPTG
6. Sucrose extract of pelleted cells
7. periplasmic extract from sucrose pelleted cells
8. supernatant from 65 °C heated then chilled cells
9. pelleted cells from 65°C heated then chilled cells
10. supernatant from 50% ammonium sulfate precipitation of 65 oC supernatant
11. pelleted proteins from 50% ammonium sulfate precipitation
12. supernatant from 65% ammonium sulfate precipitation
13. pelleted proteins from 65% ammonium sulfate precipitation
14. supernatant from 100% ammonium sulfate precipitation
15. pelleted proteins from 100% ammonium sulfate precipitation

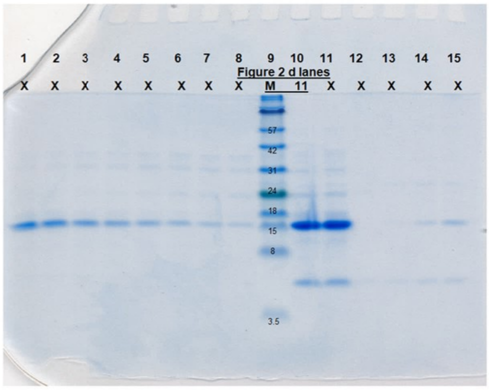

DEAE-Sephacel separation of SOD monomer
Lanes
1. fraction 42
2. fraction 43
3. fraction 44
4. fraction 45
5. fraction 46
6. fraction 47
7. fraction 48
8. fraction 49
9. BLUelf prestained protein standards
10. dialyzed 50% ammonium sulfate supernatant before separation
11. Pal Acrodisc-filtered 50% ammonium sulfate supernatant before separation
12. unbound flow through proteins
13. fraction 26
14. fraction 27
15. fraction 28

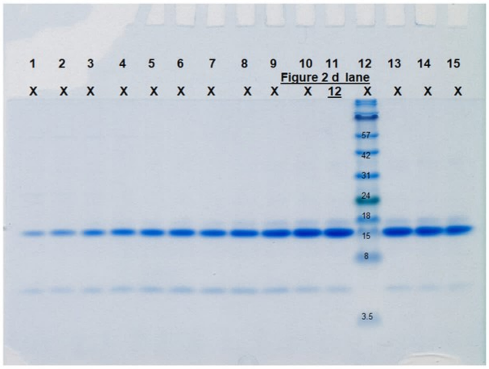

DEAE-Sephacel separation of SOD monomer
Lanes
1. fraction 29
2. fraction 30
3. fraction 31
4. fraction 32
5. fraction 33
6. fraction 34
7. fraction 35
8. fraction 36
9. fraction 37
10. fraction 38
11. fraction 39
12. BLUelf prestained protein ladder
13. fraction 40
14. fraction 41
15. fraction 42

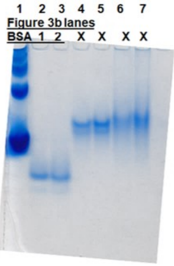

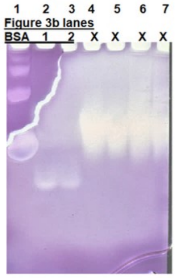

All samples were separated on one 10% NATIVE PAGE, Tris pH 8.8 without a stacking gel .
The gel was cut in half with the left gel stained with Coomassie G250.
The right gel was reacted by in-gel SOD1 enzymatic assay.
The gel was reacted with riboflavin/NBT, then TEMED and the exposed to light.
Clear bands result where SOD1 was active.

Lanes for both gels are identical

1. BSA as a standard
2. purified SOD monomer without added Cu^{2+} or Zn^{2+}
3. purified SOD monomer with added Cu^{2+} or Zn^{2+}
4. purified SOD1 dimer (the 4 mutations at interface restored) without added Cu^{2+} or Zn^{2+}
5. purified SOD1 dimer (the 4 mutations at interface restored) with added Cu^{2+} or Zn^{2+}
6.purified His6X-SOD1 dimer without added Cu^{2+} or Zn^{2+}
7. purified His6X-SOD1 dimer with added Cu^{2+} or Zn^{2+}
